# Use of Recombinant Activated Factor VII in Bleeding Lung Transplant Patients Undergoing Perioperative ECMO Therapy †

**DOI:** 10.3390/jcm12083020

**Published:** 2023-04-21

**Authors:** Daniel Laxar, Eva Schaden, Marion Wiegele, Konrad Hötzenecker, Stefan Schwarz, Johannes Gratz

**Affiliations:** 1 Department of Anaesthesia, Intensive Care Medicine and Pain Medicine, Medical University of Vienna, Spitalgasse 23, 1090 Vienna, Austria; 2 Ludwig Boltzmann Institute Digital Health and Patient Safety, Währinger Straße 104/10, 1090 Vienna, Austria; 3 Department of Thoracic Surgery, Medical University of Vienna, Spitalgasse 23, 1090 Vienna, Austria

**Keywords:** extracorporeal membrane oxygenation, Factor VIIa, hemorrhage, lung transplantation, thrombosis

## Abstract

Background: Hemostasis in critically ill patients represents a fragile balance between hypocoagulation and hypercoagulation, and is influenced by various factors. Perioperative use of extracorporeal membrane oxygenation (ECMO)—increasingly used in lung transplantation—further destabilizes this balance, not least due to systemic anticoagulation. In the case of massive hemorrhage, guidelines recommend considering recombinant activated Factor VII (rFVIIa) as an ultima ratio treatment only after several preconditions of hemostasis have been established. These conditions are calcium levels ≥ 0.9 mmol/L, fibrinogen levels ≥ 1.5 g/L, hematocrit ≥ 24%, platelet count ≥ 50 G/L, core body temperature ≥ 35 °C, and pH ≥ 7.2. Objectives: This is the first study to examine the effect of rFVIIa on bleeding lung transplant patients undergoing ECMO therapy. The fulfillment of guideline-recommended preconditions prior to the administration of rFVIIa and its efficacy alongside the incidence of thromboembolic events were investigated. Methods: In a high-volume lung transplant center, all lung transplant recipients receiving rFVIIa during ECMO therapy between 2013 and 2020 were screened for the effect of rFVIIa on hemorrhage, fulfillment of recommended preconditions, and incidence of thromboembolic events. Results and Discussion: Of the 17 patients who received 50 doses of rFVIIa, bleeding ceased in four patients without surgical intervention. Only 14% of rFVIIa administrations resulted in hemorrhage control, whereas 71% of patients required revision surgery for bleeding control. Overall, 84% of all recommended preconditions were fulfilled; however, fulfillment was not associated with rFVIIa efficacy. The incidence of thromboembolic events within five days of rFVIIa administration was comparable to cohorts not receiving rFVIIa.

## 1. Introduction

In perioperative and critically ill patients, the complex hemostatic balance between both endogenous and pharmacologically induced procoagulation and anticoagulation is particularly challenged. As such, it is affected by several factors, such as various cell types, proteins, and environmental factors [1]. Together with the tissue factor, factor VII plays a vital role in the initiation of coagulation [1]. Activated factor VII forms an integral part of the positive feedback loop that leads to the so-called thrombin burst. The administration of recombinant activated factor VII (rFVIIa) has gained increasing interest as a therapeutic option in cases of intractable bleeding. rFVIIa is an approved treatment option for acquired hemophilia A and B, congenital Factor VII deficiency, and Thrombasthenia Glanzmann—a platelet centered coagulopathy—as well as severe post-partum hemorrhage when conventional treatment fails [2].

In lung transplant recipients, the use of extracorporeal membrane oxygenation (ECMO) has become an indispensable tool for perioperative management. Particularly in patients undergoing lung transplantation due to pulmonary hypertension and cystic fibrosis, ECMO therapy is often employed [3]. When indicated, ECMO therapy may be utilized throughout the perioperative context in the preoperative (i.e., bridge-to-transplant), intraoperative, and postoperative (e.g., prolonged ECMO) periods of lung transplant recipients [4,5]. Compared with the conventional technique of transplanting lungs utilizing cardiopulmonary bypass, the intra- and postoperative use of veno-arterial ECMO therapy has been shown to improve survival in this cohort [6].

However, ECMO therapy further shifts the delicate hemostatic balance of perioperative and critically ill patients toward an increased risk of bleeding as well as thrombosis. Altered platelet function, acquired von Willebrand syndrome, and the consumption of coagulation factors may contribute to a hypocoaguable state during ECMO [7,8]. Conversely, turbulent flow, stasis, and contact of the blood with negatively charged extracorporeal surfaces increase the likelihood of thrombosis [7,8]. Thus, during ECMO therapy, systemic anticoagulation is regularly employed to counteract the increased activation of the hemostatic system; however, this further increases the risk of bleeding complications [7,8]. Bleeding events occur in 23% of ECMO patients and are associated with higher mortality [9]. When used in the perioperative context of lung transplantation, bleeding occurs in up to 39% of patients [10]. On the other hand, thromboembolic events during ECMO therapy occur in up to 25% of patients [9]. This highlights the challenged hemostatic balance during perioperative ECMO therapy, especially in the context of lung transplantation [9].

To date, no specific guidelines exist for the management of major hemorrhage during ECMO therapy. Hence, the clinical management of bleeding during perioperative ECMO support in lung transplant patients is commonly based on guidelines from other perioperative settings. The guideline on the management of severe perioperative bleeding issued by the European Society of Anaesthesiology [11] and the European Guideline on Management of Major Bleeding and Coagulopathy Following Trauma [12] mention the off-label use of rFVIIa as an ultima ratio treatment option in cases of intractable bleeding. Both guidelines recommend restoring several preconditions for hemostasis before the administration of rFVIIa [11,12]. Indeed, multiple studies in severely injured patients have investigated factors associated with rFVIIa efficacy and patient mortality. It has been shown that higher fibrinogen levels, higher hemoglobin, higher platelet count, higher temperature, and higher pH were associated with a higher efficacy of rFVIIa use and decreased mortality [10,11,12].

However, data on the efficacy and safety of perioperative rFVIIa administration are scarce, particularly in patients undergoing ECMO therapy. This study aimed to retrospectively investigate lung transplant patients on perioperative ECMO support who received rFVIIa for intractable bleeding. Specifically, we evaluated (i) the efficacy of rFVIIa in terms of bleeding control, (ii) the fulfillment of recommended preconditions before rFVIIa administration, and (iii) the occurrence of thromboembolic events following rFVIIa administration.

## 2. Materials and Methods

The present study is an investigator-initiated, retrospective, observational study performed at the Department of Anaesthesia, Intensive Care Medicine and Pain Medicine at the Medical University of Vienna, in cooperation with the Department of Thoracic Surgery, a high-volume center for lung transplantation that has conducted 498 transplantations over the last five years. Approval was obtained from the ethics committee of the Medical University of Vienna (EK 2218/2020) who waived the need for obtaining informed consent due to the retrospective nature of the study.

All patients who underwent lung transplantation at our center between August 2013 and September 2020 were screened for perioperative ECMO use and concurrent administration of rFVIIa (NovoSeven^®^ RT, Novo Nordisk A/S, Baagsvaerd, Denmark). Perioperative ECMO was defined as the uninterrupted period of ECMO therapy immediately before and/or after lung transplantation. Changes in ECMO configuration (peripheral to central and veno-venous to veno-arterial) were not considered as an interruption of ECMO therapy. At our center, ECMO therapy is initiated with a bolus dose of unfractionated heparin (UFH) and is maintained with either UFH or twice-daily low-molecular weight heparin (LMWH). As per institutional standard, heparin coated ECMO circuits were used in all included patients.

Demographics, laboratory results, and vital signs, as well as ECMO data, lung transplant indications, and medication, were extracted from the electronic health records (IntelliSpace Critical Care and Anaesthesia, Philips Healthcare, Amsterdam, Netherlands). Physician and nursing notes, radiology reports, and surgical reports were screened for thromboembolic events and bleeding episodes. Bleeding episodes were classified as (i) surgical bleeding with a localized origin of bleeding and (ii) diffuse bleeding without a localized origin of bleeding by a senior intensivist. Notes and reports were further screened for endovascular embolization or coiling.

**Figure 1:**
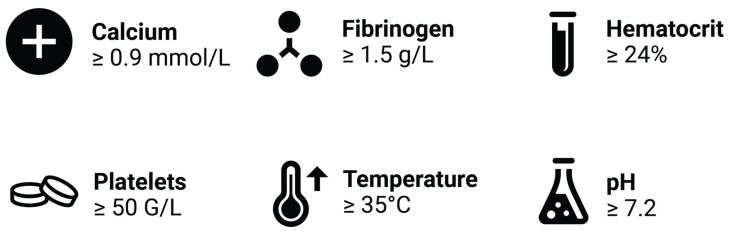
Recommended preconditions of hemostasis, as described by Kozek-Langenecker et al. [11] and Spahn et al. [12].

The current guidelines discuss the fulfillment of several preconditions prior to the administration of rFVIIa in bleeding patients. Among these are the preconditions depicted in Figure 1: calcium ≥ 0.9 mmol/L, fibrinogen ≥ 1.5 g/L, hematocrit ≥ 24%, platelet count ≥ 50 G/L, temperature ≥ 35.0 °C, and pH ≥ 7.2. In line with published literature, local standard operating procedures for critical bleeding require these preconditions to be fulfilled prior to considering rFVIIa as an ultima ratio treatment in severe bleeding. However, ultimately, the decision to administer rFVIIa is at the discretion of the treating intensivist/anesthesiologist in consultation with the treating thoracic surgeon.

Fulfillment of the recommended preconditions was investigated based on the extracted data. For each precondition, the latest measurement before the administration of rFVIIa was considered for analysis. Measurements obtained more than 12 h before administration were excluded. If multiple measurements of one parameter were available at the same time, the higher value was used for analysis. On this basis, we calculated the percentage of fulfilled preconditions prior to rFVIIa administration.

Transfusion of packed red blood cells, transfusion of platelet concentrates, administration of fibrinogen, and infusion of calcium were considered effective interventions for low hematocrit, low platelet count, low fibrinogen levels, and low blood calcium levels, respectively. If any of these interventions were detected between the last available measurement and the administration of rFVIIa, the respective precondition was considered to be fulfilled. For temperature and pH, no interventions were considered for analysis.

We dichotomously defined bleeding cessation based on clinical notes, reduction in transfused blood products, and the absence of additional administrations of rFVIIa and subsequent surgeries for hemorrhage control.

According to the data distribution, patient characteristics were presented as number, percentage, mean and standard deviation (SD), or median and interquartile range (IQR). We describe the efficacy of rFVIIa in terms of bleeding control. The effect of condition fulfillment on bleeding control was tested using Fisher’s exact test. The occurrence of thromboembolic events within five days was described. A *p*-value < 0.05 was considered statistically significant. Analysis was performed using pandas 1.3.4 and Python 3.9.

## 3. Results

### 3.1. Study Population

During the study period, 362 lung transplant patients underwent perioperative ECMO therapy at our center and were screened for the administration of rFVIIa. We excluded 341 patients who did not receive rFVIIa and 4 patients in which rFVIIa was not administered during ECMO therapy. Seventeen lung transplant patients received a total of 50 rFVIIa administrations during perioperative ECMO support. Table 1 summarizes the characteristics of the 17 included patients. Clinical data of individual cases can be found in Table 2. All 17 patients experienced clinically significant intrathoracic bleeding during or directly after either pulmonary endarterectomy or lung transplantation. According to the standard of care at our center, most patients (88%) were anticoagulated using LMWH, whereas two patients were anticoagulated using UFH during ECMO therapy [13]. Anticoagulation was paused for a median duration of 23 h for LMWH and a median duration of 4 h for UFH before rFVIIa administration. Veno-arterial ECMO was the most common ECMO configuration (15 patients, 88%); 59% of the patients underwent ECMO therapy both prior to and after lung transplantation, 35% underwent prolonged ECMO therapy after lung transplantation, which was established intraoperatively, and one patient underwent isolated preoperative ECMO therapy. 

### 3.2. Efficacy of rFVIIa

**Figure 2:**
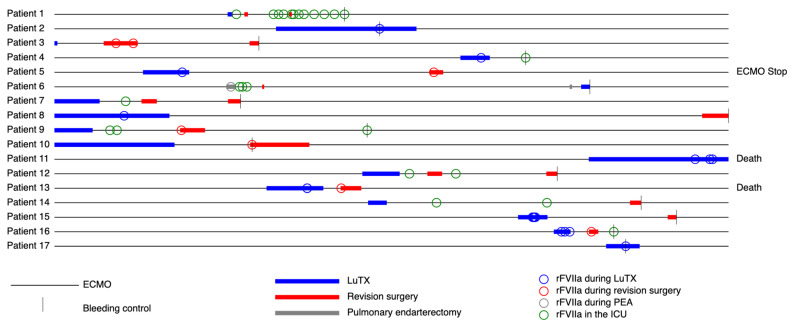
Individual administrations of rFVIIa surgeries for bleeding control depicted on a normalized ECMO timeline. The individual period of uninterrupted ECMO therapy is represented by a horizontal line for each patient. The lines are normalized over all patients, such that all lines have the same length. Horizontal bars represent surgical interventions and are colored by the type of surgery. Colored rings represent the administration of rFVIIa and vertical lines the time of bleeding control. In three patients, hemorrhage could not be controlled during ECMO therapy. Of these, one could be weaned off ECMO therapy and two patients succumbed (one in the OR, one in the ICU). ECMO = extracorporeal membrane oxygenation; rFVIIa = recombinant, activated Factor VII; LuTX = lung transplantation; PEA = pulmonary endarterectomy; ICU = intensive care unit.

Bleeding ceased after a single administration of rFVIIa in 3 out of 17 patients (18%). Another patient (6%) received two doses of rFVIIa before the bleeding ceased. In contrast, 12 patients (71%) had to undergo surgical revision due to bleeding despite rFVIIa administration. In one patient (6%), intractable bleeding occurred during lung transplantation with ongoing ECMO support following preoperative bridging. This patient died due to a massive hemorrhage, despite the administration of three rFVIIa doses. Individual timings and circumstanced rFVIIa administrations as well as their effects on bleeding control are shown in Figure 2.

During the 15 surgical revisions in 12 patients, 33% of bleeding episodes were categorized as surgical bleeding with a localized origin of bleeding and 53% as diffuse bleeding without a localized origin of bleeding. For the remaining 13%, no classification was available due to missing surgical reports.

**Figure 3:**
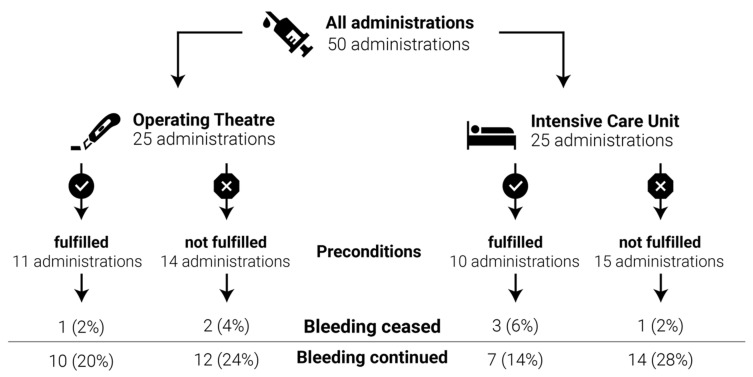
Administration of rFVIIa grouped by patients who (a) fulfilled the recommended preconditions and (b) did not fulfill the recommended preconditions in (i) the operating room and (ii) the intensive care unit.

Overall, bleeding ceased after 14% of rFVIIa administrations, whereas it continued after 86% of rFVIIa administrations. Figure 3 depicts the efficacy of rFVIIa with regard to bleeding control. Endovascular embolization or coiling was performed in none of the included patients.

### 3.3. Fulfillment of Recommended Preconditions

Eleven data points were missing for fibrinogen levels and platelet counts, and 10 data points were missing for temperature. Therefore, 12% of theoretically possible measurements were missing in our analysis.

**Figure 4:**
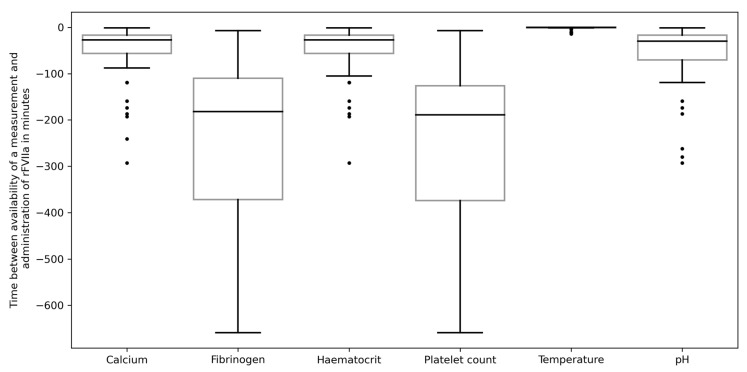
Time between the availability of a measurement and the administration of rFVIIa in minutes. Temperature was measured using a temperature catheter; calcium, hematocrit, and pH values were measured via point-of-care blood gas analysis, whereas fibrinogen levels and platelet counts were measured at the central laboratory.

The time between the availability of a measurement and the administration of rFVIIa was shorter for measurements of point-of-care tests (calcium, hematocrit, temperature, and pH) and longer for measurements obtained at the central laboratory (fibrinogen level and platelet count). The time between the availability of the measurements and rFVIIa administration is displayed in Figure 4.

**Figure 5:**
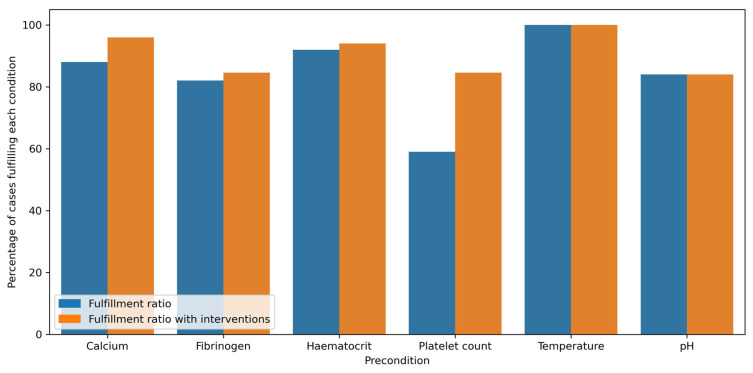
Fulfillment ratios of the recommended preconditions with and without considering interventions.

Overall, 84% of the preconditions available for rFVIIa administration were fulfilled. However, 79 interventions in 39 administrations were documented between the last measurements and rFVIIa use (11 infusions of calcium, 23 administrations of fibrinogen, 22 transfusions of packed red blood cells, and 23 transfusions of platelet concentrates). Sixty-three interventions were recorded for conditions that were already fulfilled. Assuming that all interventions were successful, a total of 91% of the conditions were fulfilled. In five administrations, all conditions were subsequently fulfilled. Figure 5 shows the individual fulfillment ratios of the recommended preconditions with and without interventions. When comparing the ratio of bleeding control after the administration of rFVIIa with the fulfillment of all recommended preconditions versus those without, we found no difference (24% vs. 12%, respectively; *p*-value: 0.43). This also held true when considering interventions: we found no difference in the ratio of bleeding control after the administration of rFVIIa after the suspected fulfillment of all recommended preconditions and without (24% vs. 9%, respectively; *p*-value: 0.42).

### 3.4. Incidence of Thromboembolic Events

In our cohort, 6 out of 17 patients experienced a thromboembolic event, four of which happened within five days after receiving rFVIIa (two pulmonary embolisms, one ischemic extremity, and one ECMO circuit clotting). One pulmonary embolism and one thrombosis of the right atrial appendage occurred 23 and 25 days after the administration of rFVIIa, respectively. All thromboembolic events were diagnosed either by radiologic imaging or—in the case of ECMO circuit clotting—clinically.

## 4. Discussion

This retrospective study is the first to systematically investigate the use of rFVIIa in bleeding lung transplant patients receiving perioperative ECMO support. Unlike previously published literature, we investigated the fulfillment of recommended hemostatic preconditions and its association with rFVIIa efficacy. We found that most of the recommended hemostatic preconditions (84%) were fulfilled before the administration of rFVIIa. Nevertheless, bleeding ceased after only 14% of administrations, and most patients (71%) had to undergo surgical revision for bleeding control following the administration of rFVIIa. None of the included patients underwent endovascular embolization or coiling.

Published data on the use of rFVIIa during ECMO support are scarce. Previously, two case reports described the use of rFVIIa in bleeding lung transplant patients receiving ECMO support: one was successful, whereas the other patient succumbed [14,15]. Repessé et al. [16] and Anselmi et al. [17] retrospectively investigated the use of rFVIIa in a mixed cohort of ECMO-supported patients. Although these studies did not specifically investigate a perioperative setting, most patients (12 out of 15 and 27 out of 30, respectively) underwent cardiothoracic surgery prior to or during ECMO therapy. Both studies suggest an acceptable efficacy and safety profile of rFVIIa in this context. 

To describe the efficacy of rFVIIa for bleeding control, we dichotomously defined whether bleeding episodes ceased. Overall, we found that only 7 out of 50 administrations of rFVIIa (14%) resulted in hemorrhage control, whereas bleeding did not cease after 43 administrations (86%). In addition, 12 out of 17 patients (71%) had to undergo surgical revision for bleeding control, in addition to the administration of rFVIIa. In contrast with our study, Repessé et al. used a mere decrease in blood drain volumes and the amount of transfused blood products to judge the efficacy of rFVIIa [16]. They reported that “bleeding dramatically decreased” after the administration of rFVIIa in 14 out of 15 patients [16]. Interestingly, active bleeding from an injured artery was identified and surgically controlled in the remaining patients. In line with our results, this finding highlights the importance of exhausting surgical options prior to the administration of rFVIIa [16]. Anselmi et al. employed a decrease in hourly chest drain output rate to assess the efficacy of rFVIIa and observed complete bleeding control in 60% of patients [17]. In contrast with our study, however, at least one surgical revision was systematically performed before the administration of rFVIIa [17].

As per institutional protocol, Repessé et al. described the following standards for the correction of hemostatic preconditions prior to the administration of rFVIIa: hematocrit ≥ 24%, prothrombin time > 50%, activated partial thromboplastin time ratio < 2, platelet count > 50 G/L, calcium > 2.2 mmol/L, fibrinogen ≥ 1 g/L, and temperature ≥ 36 °C [16]. Anselmi et al. defined the following target ranges before the use of rFVIIa: hematocrit > 24%, prothrombin time > 50%, activated partial thromboplastin time ratio < 2, platelet count > 50 G/L, fibrinogen > 1 g/L, normothermia, and normocalcemia [17]. Although the thresholds for hematocrit, platelet count, and temperature were largely comparable with our study, relevant differences in the other preconditions need to be stressed. First, in both studies, the use of thresholds for prothrombin time and activated partial thromboplastin time is advocated; however, particularly in critically ill patients, the significance of these laboratory tests has repeatedly been questioned [18,19]. None of the currently published guidelines recommend that certain prothrombin time or activated partial thromboplastin time values be met before the use of rFVIIa [11,12]. Second, Repessé et al. described the use of total serum calcium, whereas ionized calcium represents the biologically active component [20]. Third, fibrinogen is the first coagulation factor to be depleted in bleeding patients, and the fibrinogen threshold of 1 g/L used in both studies is well below the current recommendations [11,12,21]. Most importantly, in contrast with our investigation, neither study reported whether the preconditions described above were effectively fulfilled before the administration of rFVIIa [16,17].

Interestingly, fulfillment of the recommended preconditions was not associated with the efficacy of rFVIIa (i.e., bleeding control) in our study cohort. Although we observed a trend toward a higher rate of bleeding control in patients with the fulfillment of recommended preconditions versus those without (24% vs. 12%, *p*-value: 0.43), the sample sizes were too small to draw a definitive conclusion.

The administration of rFVIIa, particularly its off-label use, has been associated with thromboembolic adverse events resulting in significant morbidity and mortality [22]. Due to the high baseline risk of thromboembolic events, this is of utmost interest in patients receiving ECMO therapy. Thromboembolic events occurred in 4 out of 17 patients (24%) within the first five days after rFVIIa administration in our study. Repessé et al. reported a comparable incidence, with 3 out of 15 patients (20%) experiencing thromboembolic events following the use of rFVIIa [16]. Notably, this incidence is similar to that of patients on ECMO support without rFVIIa administration [13,23]. The 53% hospital mortality rate in our cohort is comparable to the study of Anselmi et al., who found a 30-day mortality rate of 48% [17]. Furthermore, recent studies on the Extracorporeal Life Support Organization registry showed that mortality during ECMO therapy with concomitant bleeding complications lies between 46% and 66% [9,24].

The relevant limitations of our study need to be recognized. First, the retrospective nature of our study introduces a design-inherent risk for bias, not least due to missing data points and the lack of a priori defined outcomes. Moreover, due to the retrospective design, we were not able to elucidate the decision processes leading to the individual use of rFVIIa. Second, the sample size of our study is relatively small, which does not provide adequate power to draw definite clinical conclusions, for example, with regards to the influence of precondition fulfillment on efficacy of rFVIIa. Third, we investigated a highly selected patient cohort, which might have restricted the generalizability of our results. Despite these important limitations, we are convinced that our results provide additional important and potentially hypothesis-generating information. Further research is warranted to explore the association between hemostatic preconditions and the efficacy of rFVIIa in patients with perioperative ECMO therapy.

## 5. Conclusions

In this retrospective analysis, we found that only 14% of rFVIIa administrations resulted in bleeding control, whereas surgical revision was necessary in 71% of patients. Furthermore, we found no association between the fulfillment of recommended preconditions prior to the administration of rFVIIa and subsequent bleeding control. Our data highlight the importance of exhausting surgical options for bleeding control prior to the ultima ratio use of rFVIIa.

## Figures and Tables

**Table 1:** Characteristics of the included patients. no. = number of patients; SD = standard deviation; μg = micrograms; kg = kilograms; cm = centimeters; BMI = body mass index; CTEPH = chronic thromboembolic pulmonary hypertension; pRBC = packed red blood cells; LOS = length of stay; LuTX = lung transplantation; ECMO = extracorporeal membrane oxygenation; U = unit; IU = international units; rFVIIa = recombinant, activated Factor VII.

Characteristic	Value
n	17
Age, mean (SD)	38.71 (14.22)
Sex = M, %	64.71%
Weight [kg], mean (SD)	63.35 (18.58)
BMI, mean (SD)	21.35 (5.08)
LuTX Indication, no. (%)	
Cystic Fibrosis	4 (23.53%)
Primary Pulmonary Hypertension	4 (23.53%)
Chronic Lung Allograft Dysfunction	3 (17.65%)
Interstitial Lung Disease	3 (17.65%)
CTEPH	2 (11.76%)
Primary Ciliary Dyskinesia	1 (5.88%)
Transfusions during ECMO, mean (SD)	
pRBC [U]	24.29 (16.48)
Platelet Concentrates [U]	5.88 (7.11)
Fresh Frozen Plasma [U]	18.35 (19.12)
Prothrombin Complex Concentrates [IU]	6029.41 (3882.94)
ICU LOS, mean (SD)	48 days (30 days)
ICU Death, no. (%)	8 (47.06%)
Hospital LOS, mean (SD)	57 days (32 days)
Hospital Mortality, no. (%)	9 (52.94%)
rFVIIa dose μg/kg, mean (SD)	81.59 (35.44)

**Table 2:** Clinical data of individual cases. Each row lists baseline and outcome data of an individual patient. The number of administered doses of rFVIIa in the ICU and OR as well as the cumulative dose are shown alongside the number of pRBC units transfused during ECMO therapy. Thromboembolic events occurring >5 days after the first dose of rFVIIa are printed in *italics*. ECMO = extracorporeal membrane oxygenation; LuTX = lung transplantation; OR = Operating room; ICU = intensive care unit; rFVIIa = recombinant, activated Factor VII; mg = milligram; pRBC = packed red blood cells; U = unit; LOS = length of stay; M = male; F = female; CTEPH = chronic thromboembolic pulmonary hypertension; SFA = superior femoral artery.

				rFVIIa						
Patient	Age	Sex	LuTX-Indication	OR	ICU	Dose [mg]	pRBC [U]	Thromboembolic Event	ICU LOS [Days]	ICU Death
**1**	26	F	Cystic Fibrosis	0	12	60	57	-	36	yes
**2**	60	M	Interstitial Lung Disease	1	0	3	8	Right SFA occlusion	54	no
**3**	48	M	Primary Ciliary Dyskinesia	2	0	10	29	-	80	yes
**4**	31	M	CTEPH	1	1	10	39	Pulmonary embolism	22	yes
**5**	24	F	Cystic Fibrosis	2	0	10	15	-	106	no
**6**	58	M	CTEPH	1	3	20	60	ECMO circuit clotting	42	yes
**7**	38	M	Primary Pulmonary Hypertension	0	1	5	21	*Thrombosis of the right atrial appendage*	54	yes
**8**	40	M	Primary Pulmonary Hypertension	1	0	10	16	-	34	no
**9**	26	F	Primary Pulmonary Hypertension	1	3	15	25	-	31	no
**10**	57	M	Interstitial Lung Disease	1	0	5	5	-	106	no
**11**	37	F	Primary Pulmonary Hypertension	3	0	15	5	-	4	yes
**12**	33	F	Cystic Fibrosis	0	2	7.2	27	*Pulmonary embolism*	75	no
**13**	34	M	Chronic Lung Allograft Dysfunction	3	0	15	19	Pulmonary embolism	6	yes
**14**	66	M	Interstitial Lung Disease	0	2	10	35	-	73	no
**15**	19	F	Cystic Fibrosis	4	0	13	16	-	30	no
**16**	36	M	Chronic Lung Allograft Dysfunction	4	1	20	30	-	47	yes
**17**	25	M	Chronic Lung Allograft Dysfunction	1	0	5	6	-	33	no

## Data Availability

Data will be made available to interested parties upon reasonable request. Public availability is not feasible due to local policies.
